# Effectiveness of Computer Tailoring Versus Peer Support Web-Based Interventions in Promoting Physical Activity Among Insufficiently Active Canadian Adults With Type 2 Diabetes: Protocol for a Randomized Controlled Trial

**DOI:** 10.2196/resprot.5019

**Published:** 2016-02-11

**Authors:** François Boudreau, Michel Moreau, José Côté

**Affiliations:** ^1^ Interdisciplinary Group of Health Applied Research Department of Nursing Université du Québec à Trois-Rivières Trois-Rivières, QC Canada; ^2^ Research Centre of the Centre Hospitalier de l'Université de Montréal Faculty of Nursing Université de Montréal Montréal, QC Canada

**Keywords:** physical activity, behavior modification, computer tailoring, personalization, Facebook, peer support, Internet, World Wide Web, eHealth, diabetes mellitus, Type 2, motor activity, behavior therapy, self-help groups, telemedicine

## Abstract

**Background:**

Type 2 diabetes is a major challenge for Canadian public health authorities, and regular physical activity is a key factor in the management of this disease. Given that less than half of people with type 2 diabetes in Canada are sufficiently active to meet the Canadian Diabetes Association's guidelines, effective programs targeting the adoption of regular physical activity are in demand for this population. Many researchers have argued that Web-based interventions targeting physical activity are a promising avenue for insufficiently active populations; however, it remains unclear if this type of intervention is effective among people with type 2 diabetes.

**Objective:**

This research project aims to evaluate the effectiveness of two Web-based interventions targeting the adoption of regular aerobic physical activity among insufficiently active adult Canadian Francophones with type 2 diabetes.

**Methods:**

A 3-arm, parallel randomized controlled trial with 2 experimental groups and 1 control group was conducted in the province of Quebec, Canada. A total of 234 participants were randomized at a 1:1:1 ratio to receive an 8-week, fully automated, computer-tailored, Web-based intervention (experimental group 1); an 8-week peer support (ie, Facebook group) Web-based intervention (experimental group 2); or no intervention (control group) during the study period.

**Results:**

The primary outcome of this study is self-reported physical activity level (total min/week of moderate-intensity aerobic physical activity). Secondary outcomes are attitude, social influence, self-efficacy, type of motivation, and intention. All outcomes are assessed at baseline and 3 and 9 months after baseline with a self-reported questionnaire filled directly on the study websites.

**Conclusions:**

By evaluating and comparing the effectiveness of 2 Web-based interventions characterized by different behavior change perspectives, findings of this study will contribute to advances in the field of physical activity promotion in adult populations with type 2 diabetes.

**Trial Registration:**

International Standard Randomized Controlled Trial Number (ISRCTN): ISRCTN15747108; http://www.isrctn.com/ISRCTN15747108 (Archived by WebCite at http://www.webcitation.org/6eJTi0m3r)

## Introduction

### Background

In 2009, 2.4 million Canadians were living with type 2 diabetes (T2D), and this number is expected to grow to 3.7 million by 2019 [1]. Scientific evidence increasingly shows the importance of regular moderate-intensity aerobic physical activity (PA) as a means of reducing the risk of complications associated with T2D [2-4]. Despite this body of evidence, self-reported measures suggest that only 30 to 40% of Canadians with T2D are meeting the Canadian Diabetes Association's guidelines [5,6]. Accordingly, innovative strategies aimed at promoting the adoption and maintenance of regular PA among Canadians with T2D are needed [1], and information technologies such as the Internet are a promising avenue for the development of large-scale interventions with behavior change purposes [7-11]. The evolution of Canadian Internet use also suggests that public health organizations should take advantage of this technology to develop and deliver services [12-14], including services targeting populations with chronic diseases [15] and adults ranging from 18 to 65 years of age [16-18].

To date, a significant number of scientific reviews have studied the effectiveness of Web-based interventions designed for PA adoption and/or maintenance, with evidence showing that they can be effective across various populations and settings [19-24]. However, only a few studies have attempted to develop Web-based PA-oriented interventions specifically for people with T2D, which have only produced mixed results so far [25-27]. Among these studies, the most successful interventions have been characterized by the following [25,27]: (1) they were theory-based; (2) they included goal-setting activities; (3) they included interactive tracking and personalized feedback components; and (4) they provided opportunities for peer support. In line with these insights, two web-based interventions using different behavior change perspectives will be evaluated and compared in this study regarding their effectiveness in promoting the adoption and maintenance of regular moderate-intensity aerobic PA in adults with T2D.

The first intervention consists of a fully automated, computer-tailored, Web-based intervention developed from the perspective of offering tracking and personalized feedback. In eHealth research, the technology underlying the tracking and personalized feedback component of interventions is known as “computer tailoring,” which can be defined as the generation of personalized feedback by a computer program based on prior individual assessment(s) [28]. This technology follows principles of face-to-face counselling and assesses individuals’ perceived health behavior status as well as the determinants that influence their motivation and behavior. Next, tailored feedback is provided about these determinants based on individuals’ answers and personal characteristics. According to the Elaboration Likelihood Model [29], the provision of tailored feedback will result in more thoughtful information processing via the central route of persuasion, since tailored messages are perceived as being personally relevant and thus encourage the person to pursue the desired behavior [30]. The technology of computer tailoring has proven to be more effective than more general types of health behavior change interventions, including those targeting PA adoption [7,31-33]. Yet, to our knowledge, only a few studies to date have used computer-tailoring technologies for the purpose of promoting regular moderate-intensity aerobic PA among adults with T2D [34-36].

The second intervention consists of a peer support, Web-based intervention that was developed from the perspective of offering Web-based opportunities for peer support. Within a health care context, peer support is defined as “the provision of emotional, appraisal, and informational assistance by a created social network member who possesses experiential knowledge of specific behavior or stressor and similar characteristics as the target population, to address a health-related issue of a potentially or actually stressed focal person” [37]. Social networking sites, of which Facebook is the most popular platform [38], can offer an innovative way of delivering peer support interventions to promote the adoption of health-related behaviors [39]. In this regard, the use of Facebook as a means of offering opportunities for peer support for promoting PA is relatively new. Evaluative studies published to date demonstrate the potential of this platform among young adult cancer survivors [40], healthy adults [41], female undergraduate students [42], female college freshmen [43], and government employees with metabolic syndrome [44]. Yet, to our knowledge, no study to date has used this platform for the purpose of enabling adults with T2D to share their experiences, concerns, and progress with their peers regarding the adoption of regular moderate-intensity aerobic PA specifically.

A substantial body of evidence stresses the importance of using a theoretical framework to inform the development of behavior change interventions [7,25,45]. The development of both interventions was thus based on the I-Change Model [46]. The I-Change Model is a theoretical framework that integrates the main concepts of several social cognitive theories (eg, Theory of Planned Behavior [47], Social Cognitive Theory [48], Health Belief Model [49], and the Transtheoretical Model [50]). The I-Change Model distinguishes 3 phases and their corresponding determinants in the behavior change process. For instance, to promote regular moderate-intensity aerobic PA in adults with T2D, an intervention should (1) increase a person’s awareness about the importance of practicing moderate-intensity aerobic PA; (2) motivate the person to practice moderate-intensity aerobic PA; and (3) transfer this motivation to the adoption of regular moderate-intensity aerobic PA [51]. Furthermore, the I-Change Model assumes that the determinants of the 3 phases mentioned above are affected by, among other influences, channel factors, which refer to the methods used to deliver messages. In this research project, the computer-tailoring component and peer support component are considered to be two different channels for message delivery. It is hypothesized that these channels will foster the adoption of regular moderate-intensity aerobic PA through cognitive changes (eg, attitude, social influence, self-efficacy, intention) [37,52].

In addition, to explore a current trend in Web-based interventions with health behavior change purposes [53-58], concepts derived from the Self-Determination Theory (SDT) [59] and Motivational Interviewing (MI) [60] are added to the I-Change Model as elements of the theoretical framework. SDT is a theory focusing on types of motivation rather than solely on the amount of motivation [59], while MI is a counseling approach using a collaborative style of communication to strengthen motivation and commitment to change in individuals [60]. As it has the potential to foster more autonomous motivation toward adopting and maintaining health behaviors in individuals [61], the integration and application of SDT and MI into behavior change interventions have been extensively discussed and encouraged in the PA domain in recent years [62-68]. Recent positive findings from a randomized controlled trial highlighted that more research on the effects of SDT and MI in Web-based PA behavior change interventions is needed [69].

### Aim of the Study and Hypotheses

The primary aim of this study is to evaluate the independent effectiveness of two Web-based interventions in promoting regular moderate-intensity aerobic PA among insufficiently active adult Canadian Francophones with T2D. In line with this aim, it is hypothesized that the Web-based computer-tailored intervention (H1) and the Web-based peer support intervention (H2) will be more effective at increasing moderate-intensity aerobic PA levels compared to a control group at 3 and 9 months after baseline. Another aim of this study is to compare the relative effectiveness of the two Web-based interventions at increasing moderate-intensity aerobic PA levels. Hence, the third hypothesis is that participants in both intervention groups will show a different level of moderate-intensity aerobic PA at 3 and 9 months after baseline. This hypothesis is formulated bilaterally, because no study to date has compared these two methods.

## Methods

### Design

The study design is a parallel, randomized controlled trial (RCT) with the following 3 study arms: (1) an experimental group receiving an 8-week, fully automated, computer-tailored, Web-based intervention; (2) an experimental group receiving an 8-week, peer support, Web-based intervention; and (3) a control group that does not receive any intervention during the study period. A baseline measurement (T0) and two follow-up measurements at three (T1) and nine (T2) months after baseline are included. All three arms follow the same study timeline and are invited to complete the assessments during the same period. The protocol was developed in accordance with the CONSORT-EHEALTH checklist [70] and the SPIRIT statement 2013 [71].

### Eligibility and Recruitment

The target population for this research project is Canadian men and women with self-reported T2D residing in the province of Quebec. Other inclusion criteria to be eligible for the research project are not meeting the Canadian Diabetes Association guidelines on moderate-intensity aerobic PA [72], being able to understand French, having access to the Internet, being between 18 and 65 years of age, and not having medical indications limiting the practice of PA. Eligibility was evaluated with a self-reported questionnaire administered to potential participants via the intervention websites during the study registration process.

As for the recruitment process, the research project was implemented in partnership with Diabète Québec, a recognized association for people with T2D in the province of Québec, Canada. Following a joint agreement between both parties, Diabète Québec sent 2 invitation emails over a 2-week period (from 2014 September 15 to 28) to all Canadian Francophones with T2D meeting the age criteria and who subscribed to the association’s newsletter.

### Randomization

Two websites were created for this study: one to host activities related to the computer-tailored intervention, and the other to host activities for the peer support intervention and those of the control group. Randomization with a 1:1:1 allocation ratio was performed in three steps. First, all Diabète Québec newsletter recipients with T2D who were between 18 and 65 years of age received invitation emails to participate in the study, resulting in 6425 potential participants. Next, 35% of these 6425 potential participants were randomly sampled by Diabète Québec via a computer algorithm to receive emails with a URL link redirecting them to the first website (computer tailoring group). Meanwhile, the remaining 65% of potential participants received emails giving them access to a URL redirecting them to the second website. Finally, potential participants who had been redirected to the second website were then allocated randomly by the web solution company responsible for the website development to either the peer support intervention group or the control group via a computer algorithm. The randomization procedures mentioned above were conducted independently of the research team. This is an open-label RCT, meaning that participants and investigators know who has received which intervention.

### Sample Size Calculation

Calculation of the sample size was achieved with GPower 3.0.10, and instructions for selecting different criteria were taken from Erdfelder et al [73]. Thus, according to a mixed-plan, 3 (2 experimental groups and 1 control group) x 3 (baseline and 3- and 9-month follow-ups) design, assuming a statistical power of .80 and an alpha value of .05, a total sample size of 204 participants is necessary to detect a conservative effect size of d=.20 [74]. This effect size was chosen with reference to results observed in the field of computer-tailored interventions [33,75] and considering the modest evidence observed to date for Web-based interventions integrating online opportunities for peer support [25,76]. Moreover, to obtain the calculated sample size at the end of the study (N=204), it was estimated that at least 6000 participants needed to be solicited through the invitation emails sent by Diabète Québec. This estimate was based on a similar study [77], and a review of additional literature: (1) from the 6000 participants solicited, 30% (1800/6000) will agree to participate in the study [77,78]; (2) of this number, almost 100% (1800/1800) will have T2D, since preselection of potential participants with this type of diabetes will be executed by Diabète Québec; (3) 85% (1530/1800) will not have medical indications limiting the practice of moderate-intensity aerobic PA [79]; (4) 70% (1071/1530) will not meet the Canadian Diabetes Association guidelines related to aerobic PA [77]; (5) 70% (749/1071) will have access to the Internet [15]; and (6) 60% (449/749) will complete the study [77]. However, this algorithm has overestimated the number of participants enrolled at baseline, which was 234.

### Interventions

**Figure 1:**
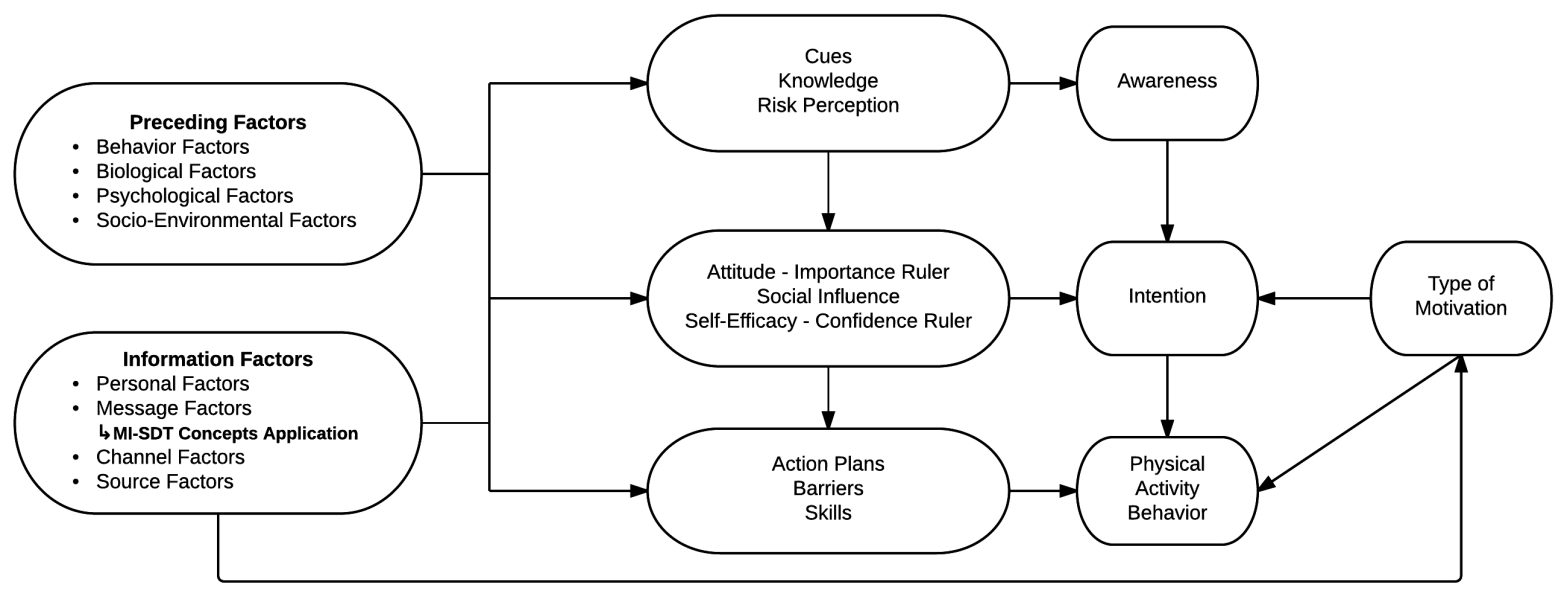
The theoretical framework of both interventions integrating the I-Change Model, Self-Determination Theory (SDT), and Motivational Interviewing (MI).

The Web-based computer-tailored intervention and the Web-based peer support intervention followed a rigorous theory-based and evidence-based development process involving a literature review, focus groups with T2D patients, a usability testing phase, and multidisciplinary expertise. As outlined in the background section, both interventions are based on the I-Change Model, in which concepts borrowed from SDT and the MI approach are integrated as part of the theoretical framework (Figure 1). These concepts are as follows: (1) type of motivation (SDT-related); (2) importance ruler (MI-related); (3) confidence ruler (MI-related); and (4) MI-SDT concepts application. In short, feedback messages about the *type of motivation* aim to inform participants in both interventions that controlled or autonomous forms of motivation can influence the adoption and maintenance of regular PA [80]. *Importance ruler* and *confidence ruler* are used mainly to provide feedback to participants with MI-related strategies. The remaining added construct, *MI-SDT concepts application*, aims to provide messages to participants using a style of communication that is consistent with both SDT and MI across all of the interventions’ features.

### Content Specific to the Computer-Tailored Intervention

A key component of the computer-tailored intervention is that it uses recent techniques borrowed from computer-tailoring research to provide messages in a personalized fashion to its participants [81,82]. Eight computer-tailored motivational sessions based on PA-related cognitions and current PA level were offered to participants. These sessions provided messages matching how individual participants scored on each cognitive construct and were complemented with personalization (eg, name) and different types of feedback (eg, descriptive, comparative, or evaluative) [81]. One motivational session per week was made available to participants over an 8-week period. The order in which each of these motivational sessions was offered in the intervention was established so as to fit the theoretical framework and its 3 related phases. First, motivational session 1 aimed to raise awareness among participants regarding how their insufficient PA level could impact their health (ie, tailored feedback on risk perception and current PA level). This corresponds to the first phase of our theoretical framework: increasing the importance of change in participants [83]. Next, all other sessions aimed to motivate participants toward increasing their PA level over the course of the intervention by providing them with tailored feedback related to their attitude, social influence, self-efficacy, type of motivation, and intention. This corresponds to the second phase of our theoretical framework, which was to develop participants’ motivation for making the desired change [84]. Moreover, all computer-tailored motivational sessions were built such that they ended by querying participants about their desire to build a PA action plan for the week, which aimed to help them take action, and thus, increase their PA level. For instance, if a participant told the software he wanted to build an action plan, he was redirected to the action planning tool available on the website to build his plan. This last step is in concordance with the final phase of our model, in which motivation is transferred into the adoption of the desired behavior [85]. To integrate SDT and MI within the computer-tailoring component, all motivational sessions were also interactively geared so that participants could develop an autonomous motivation to practice more PAs. Furthermore, all sessions used various techniques associated with MI and its basic interview skills: (1) asking open questions, (2) affirming, (3) reflecting, (4) summarizing, and (5) informing and advising [60] (see Multimedia Appendix 1) [86-88]. Finally, readers are encouraged to read Moreau et al [80] for an in-depth description of the algorithms behind the tailoring component of the intervention and examples of messages received by participants during computer-tailored motivational sessions.

### Content Specific to the Peer Support Intervention

A key component of the peer support intervention is that it provides opportunities for participants to support each other by using a Web-based discussion group. More precisely, the online peer support component can be viewed as a semi-guided private Facebook group moderated by a clinical nurse. At their convenience, participants had the opportunity to visit either the intervention website or to interact with other participants via the Facebook group during an 8-week intervention period. Participants randomized into the peer support intervention were exposed to the same content as participants in the computer-tailored intervention regarding PA-related cognitions. However, the peer support intervention was targeted, meaning that the same content was provided to all participants, regardless of their individual characteristics beyond being adults with T2D. For the first 4 weeks, participants received 2 web articles per week that provided information on PA-related topics (eg, risk perception, PA guidelines, attitude), which were made available directly on the website and posted simultaneously in the Facebook group. For weeks 5, 6, and 7, participants received 1 web article per week that provided information on other PA-related topics (eg, self-efficacy, intention, and action planning). No article was offered during the last week, allowing the nurse-moderator to discuss participants’ appreciation for the intervention through the Facebook group. Similar to the computer-tailored intervention, the order in which each web article was made available was established to correspond to the theoretical framework of the study. All topics in each web article are presented in Multimedia Appendix 2.

The clinical nurse moderated the group using a method we developed that integrates SDT concepts and MI techniques, aiming to encourage participants to focus their discussions on PA-related topics and develop their own reasons for practicing more moderate-intensity aerobic PAs [60] (see Multimedia Appendix 1). The following steps explain the method used by the moderator: (1) participants were first greeted by the nurse-moderator when they joined the Facebook group; (2) they were then invited to read the introductory text describing the underlying MI spirit sought for the group, which was accessible in the group’s description section; (3) when posting a new web article to the Facebook group’s page, the nurse-moderator always simultaneously posted 1-2 open-ended questions on the group’s wall that were related to the article topic (see Multimedia Appendix 3) and aimed to encourage participants to develop their own motivation; (4) 2 days after the questions were posted, the nurse-moderator summarized the answers posted by participants, with an emphasis on comments made in favor of adopting the desired behavior; (5) for the last 4 weeks of the intervention, since only 1 article was posted per week during this period (or no article during the last week), the nurse-moderator spent at least 1-2 hours per day, 3 days a week, replying to specific participants’ comments to show interest, encourage further self-exploration, and favor group participation [60]. Despite this standardized method adopted by the moderator, it is important to note that participants were part of a peer support intervention and most interactions in the group were between themselves. While participants had the opportunity to talk about the web articles posted on the Facebook group’s page, they could also discuss any topic or concern they wanted to share with other participants.

### Additional Features

Despite their distinct core content, both interventions possessed the following similar features: (1) the appearance of the website home page, (2) a tool for self-monitoring of PA behavior, (3) tools for goal setting and action planning, (4) tabs providing safety tips on how to practice PA properly, and (5) access to technical support via email. The designs of the websites were different except for the home page (see Multimedia Appendix 4), as they were developed by different web solution companies at different times during the study development phase. Additionally, other evidence-based behavior change techniques according to Michie et al’s taxonomy [89] were integrated into both interventions and can be viewed in detail in Multimedia Appendix 5 [90-96]. These were identified in the literature review preceding the development of both interventions [80]. Lastly, the computer-tailored and peer support interventions both started on 29 September 2014 and ended on 22 November 2014.

Table 1 presents a broad overview of the intervention components for each experimental condition. Multimedia Appendix 2 presents a more detailed overview of both interventions, including timelines.

**Table 1:** Overview of components found in each intervention group and the control group

		Study groups		
		Computer-tailored intervention	Peer support intervention	Control group
**Computer tailoring**				
	Personalization^a^	x^b^		
	Content matching^a^	x^b^		
	Feedback^a^	x^b^		
Peer support opportunities			x^b^	
**PA: cognitions messages**				
	Intention	x	x	
	Attitude (pros and cons)	x	x	
	Social influence	x	x	
	Self-efficacy	x	x	
	Risk perception	x	x	
	Type of motivation	x	x	
Action planning		x	x	
Goal setting		x	x	
Self-monitoring of PA		x	x	
Periodic email prompts^c^		x	x	x

^a^Contemporary computer-tailoring terminology summarized by Harrington and Noar [81].

^b^Distinctive components of each intervention

^c^Emails were sent only to stimulate participants to reuse the interventions or to complete posttest evaluations.

### Control Group

Participants in this group did not participate in any intervention for the duration of the study. Following the last follow-up questionnaire, participants from this group were offered the fully automated, computer-tailored, Web-based intervention.

### Outcome Assessments

Emails were used to invite participants to complete baseline, 3-month, and 9-month follow-up questionnaires on the intervention websites. Participants were invited to complete their baseline questionnaire during the enrolment period between 2014 September 15 to 2014 September 18. Participants were invited to complete their 3-month and 9-month follow-up questionnaires during the period between 2014 December 16 and 2015 January 4 and during the period between 2015 June 5 and 2015 July 4, respectively. As it is well known that Internet interventions generally suffer from a high attrition rate [97], a financial incentive was offered to participants for completing the 3-month ($20) and 9-month ($10) follow-ups.

### Primary Outcomes

PA level was evaluated with an adapted version of the Godin Leisure Time Exercise Questionnaire (GLTEQ) [98,99]. An independent evaluation of this instrument indicated that it compared favorably in terms of reliability and validity to 9 other self-report measures of PA on test-retest methods, objective measure of PA, and fitness indexes [100]. The GLTEQ had a one-month test-retest reliability of .62 and concurrent validity coefficients of .32 with an accelerometer and .56 with maximum oxygen uptake [100]. Participants were asked (1) the frequency with which they practiced intensity-specific aerobic PAs (ie, low, moderate, vigorous) in a typical 7-day week and (2) the average duration in minutes of these activities for each intensity category. Final PA levels were obtained by calculating the converted total minutes per week of moderate-intensity aerobic PA that participants engaged in during a typical week; only moderate (4 metabolic equivalents [METS]) to vigorous (7.5 METS) types of aerobic PA were considered, and the total minutes of vigorous aerobic PA/week were considered as 1.875 × minutes of moderate aerobic PA/week (7.5 METS/4 METS = 1.875). Using this method, a participant was considered “regularly active” with a score greater than or equal to 600 METs-min/week. This threshold is the equivalent of 150 min of moderate-intensity aerobic PA per week and corresponds to the recommendations of the Canadian Diabetes Association [72].

### Secondary Outcomes

PA-related cognitions were also assessed to identify the mediating processes through which behavior change occurred in participants according to the answers they provided during the baseline, 3-month, and 9-month follow-up assessments on the study’s websites. The items used for the assessment of attitude, social influence, self-efficacy/perceived behavioral control, and intention were developed according to the guidelines from Ajzen's Theory of Planned Behavior [47] (Table 2). A test-retest procedure was conducted over a 2-week period with a subgroup of 29 individuals with T2D. The mean age of the participants was 57.3 years (SD 10.8), 48% (14/29) were male, and 45% (13/29) had completed postsecondary education. The Cronbach alpha coefficients and intraclass correlation coefficients are reported in Table 2. As for the items assessing the type of motivation of participants, a modified, validated, French-language version of the BREQ-2 [101] (Behavioural Regulation in Exercise Questionnaire, version 2) was used to measure all possible types of motivation a participant could experience, including integrated motivation, which is normally absent in the standard BREQ-2 [102]. The investigators who developed the questionnaire assessed the construct and discriminant validity with confirmatory factor analysis. Nomological and convergent validity were also assessed with correlation analysis. Questionnaire validation results are reported elsewhere [101].

**Table 2:** Description of psychosocial variables and psychometric values.

Variable	Items	Scale	Internal consistency^ a ^	Test-retest reliability ^ c ^
Intention			.84	.45
	I intend to be regularly active in the next month.	Unlikely (+1)/likely (+7)		
	My plans are to practice physical activities regularly in the next month.	Disagree (+1)/agree (+7)		
	I estimate that my chances of practicing physical activities regularly over the next month are...	Weak (+1)/good (+7)		
Attitude			.58	.49
	I think that practicing physical activities regularly in the next month would be…	Bad (+1)/good (+7)		
		Useless (+1)/useful (+7)		
		Unenjoyable (+1)/enjoyable (+7)		
		Unpleasant (+1)/pleasant (+7)		
Self-efficacy			.83	.57
	I feel capable of practicing physical activities regularly in the next month.	Disagree (+1)/agree (+7)		
	For me, practicing physical activities regularly in the next month would be…	Difficult (+1)/easy (+7)		
	How much control do you feel you have over your ability to practice physical activities regularly?	No control (+1)/control (+7)		
Social influence (subjective norm)			.87^b^	.77
	People who are important to me would support me if I were to practice physical activities regularly in the next month.	Disagree (+1)/agree (+7)		
	People who are important to me think I should practice physical activities regularly in the next month.	Disagree (+1)/agree (+7)		
Social influence (descriptive norm) ^ d ^			—	—
	How many people who are part of your daily life (children, spouse/partner, physician, friends, colleagues, etc) are physically active on a regular basis?	Nobody (+1); a minority (+2); half (+3); a majority (+4); everybody (+5)		

^a^Internal consistency reported as Cronbach alpha coefficient for variables of 3 items or more.

^b^Internal consistency reported as Spearman’s correlation coefficient for variables of 2 items.

^c^Intraclass correlation coefficient.

^d^No data provided for descriptive norm, given the change of scale following the test-retest.

### Statistical Analysis

Descriptive statistics will be used to summarize the baseline characteristics of the participants for sociodemographic variables (ie, origin, occupation, gender, age, and education), anthropometric variables (ie, height, weight, and body mass index), psychosocial variables (ie, attitude, social influence, self-efficacy, type of motivation, and intention), and PA level. In line with the hypotheses, linear mixed model analyses for repeated measures will be used to examine the effectiveness of the computer-tailored intervention (H1) and peer support intervention (H2) relative to the control group and also to compare both interventions to each other (H3). A linear mixed-model approach is very well suited for modelling data containing repeated measures from several participants [103] and has the advantage of providing better capabilities for handling missing observations when data are missing at random, compared to more traditional approaches such as repeated measures analysis of variance [104,105]. In accordance with the statistical procedure and for each analysis, group, time, and time × group will be included as independent variables and minutes per week of moderate-intensity aerobic PA will be included as the dependent variable. Analyses will be adjusted for sociodemographic characteristics and other relevant variables that significantly differ between the 3 study arms at baseline. In the presence of statistically significant interaction terms, post-hoc tests will be applied to compare differences between all pairs of means. All pairwise tests of the computer-tailored or peer support interventions compared to the control group will be 1-tailed, and tests comparing both interventions together will be 2-tailed. Finally, potential mediators of the interventions’ effects will be explored. Based on the theoretical framework (Figure 1), a mediation analysis will be conducted involving a multicategorical independent variable (both interventions relative to control group), with type of motivation and intention as mediator variables in serial, and PA behavior as the dependent variable. Hayes and Preacher’s SPSS macro will be used for exploring the potential mediators [106]. The statistical analyses will be conducted with SPSS 22.0.

### Ethics Approval and Participant Consent

Ethics approval was obtained from the Ethics Committee of Research with Humans from the Université du Québec à Trois-Rivières on 2014 September 12 (CER–13–194–08.03.04). All participants in the study gave their informed consent online via the study websites during the registration process.

## Results

The project was funded in 2011 and enrolment was completed in September 2014. Data analysis is currently under way and the first results are expected to be submitted for publication in 2016.

## Discussion

The purpose of this study is to assess the effectiveness of two Web-based interventions, each of which was developed based on different behavior change perspectives, to promote regular moderate-intensity aerobic PA among adults living with T2D. Several meta-analyses of traditional T2D self-management education programs have demonstrated the effectiveness of certain lifestyle programs [107-110]. In Canada [111], as elsewhere [112,113], these programs have mostly been developed in group or individual format sessions. However, in the province of Québec, where the study is being conducted, diabetes education centers cannot meet the demand for traditional self-management education programs serving adults diagnosed with the disease, particularly in large urban regions and remote areas [114]. This observation, combined with the ever-increasing prevalence of diabetes [18,115] and the fact that still only 50% of Canadian patients with T2D meet the recommended glycated hemoglobin target of ≤ 7.0% [116], provide a clear call-to-action for the implementation of wide-reaching programs helping people with T2D to improve their lifestyles. With that purpose in mind, computer-tailored interventions and peer support interventions offered through the Internet and focusing on lifestyle changes represent two highly credible solutions that deserve attention.

The proposed study has several strengths. First, a rigorous theory-based and evidence-based intervention development process was employed. Notably, considerable efforts were devoted to base the intervention offering online peer support on an evidence-based theoretical framework, which is less frequent for this type of intervention [76]. Second, the integration of SDT and MI in a Web-based PA promotion context is innovative and has been attempted by few research teams to date. Positive results suggest that additional research should explore this path in the future [69]. Third, another strength of this study is its rigorous reporting of the interventions’ components, behavioral change techniques, and theoretical framework used; such rigor is increasingly required for comprehensiveness, reproducibility, and comparison among trials [81,110,117-119].

However, this study is not without limitations. First, given its open-label design (ie, not blinded), although quite common in Web-based interventions delivered under free-living conditions [110], participants in all 3 study groups and researchers were aware of the participants’ group assignment. This has the potential to introduce performance bias, defined as “systematic differences between groups in the care that is provided, or in exposure to factors other than the interventions of interest” [120]. All participants were, however, unaware of the hypotheses of the study and were only told that the researchers wanted to assess the effect of different Web-based interventions on PA behavior in adults with T2D. Second, given the use of self-reported measures for the outcome assessments, even from validated questionnaire items, this has the potential to introduce detection bias, defined as **“**systematic differences between groups in how outcomes are determined**”** [120]**.** However, outcome data assessment was automated through the study websites and was thus blinded to the research team, which should reduce the possibility of a differential bias between intervention groups and the control group. Furthermore, the third hypothesis regarding the comparative effectiveness of the interventions was formulated bilaterally, reducing the possibility of bias on the investigators’ part. Finally, one possible limitation across Web-based interventions is the attrition phenomenon [97,121]. Despite a smaller than expected number of participants recruited at baseline and the impossibility of fully preventing a high attrition rate, some evidence demonstrated that adding personalization, computer tailoring, and peer support components could contribute to reducing the attrition rate in Web-based interventions [110,121]. Financial compensation associated with follow-up completion might also have helped reduce the attrition rate of our study [122].

To the best of our knowledge, this trial represents one of the first studies seeking to compare a Web-based computer tailoring intervention and a Web-based peer support intervention that are both PA-focused and designed for patients with T2D. Results of this study have the potential to inform future developers about the effectiveness of PA-focused, Web-based interventions for patients with T2D, including which of the two behavior change perspectives employed should be prioritized. If positive results are observed, developers could also use information provided in this manuscript to gain insights about which additional components to include in their Web-based interventions.

## Appendix

Multimedia Appendix 1 Motivational Interviewing (MI) and Self-Determination Theory (SDT) concepts application to both interventions.

Multimedia Appendix 2 Trial overview: timeline and intervention components.

Multimedia Appendix 3 Moderator's questions: peer support intervention.

Multimedia Appendix 4 Layout of websites.

Multimedia Appendix 5 Behavior change techniques for both interventions.

## Abbreviations

DEF: Diabète en Forme
MI: motivational interviewing
PA: physical activity
SDT: self-determination theory
T2D: type 2 diabetes
