# Supplementary material for: Effectiveness of Computer Tailoring Versus Peer Support Web-Based Interventions in Promoting Physical Activity Among Insufficiently Active Canadian Adults With Type 2 Diabetes: Protocol for a Randomized Controlled Trial
Source: JMIR Res Protoc. 2016 Feb 11;5(1):e20. doi: 10.2196/resprot.5019 (PMC4768043; doi:10.2196/resprot.5019)
Supplement: Multimedia Appendix 1 [file resprot_v5i1e20_app1.pdf]

Multimedia Appendix 1. Overview of MI-SDT concepts application to both interventions: SDT needs and related need-supportive strategies.

**Autonomy: being the perceived origin or source of one's own behavior [1]**		
**MI-related strategies**		
Strategies	**Computer-tailored intervention**	**Peer-support intervention**
Let the client make decisions about what and how to change [2]	The weekly action plan tool to which participants have access allows them to select their objectives and activities by themselves. The intervention explicitly recognizes throughout its components that the participant is the best person to make decisions about what and how to change.	The fourth web article provides an action plan tool to which participants have access for the rest of the intervention, allowing them to select their objectives and activities by themselves. The intervention explicitly recognizes throughout its components that the participant is the best person to make decisions about what and how to change.
Roll with resistance [2]	The intervention has specific feedback for participants with low scores on intention, self-efficacy, and attitude. Reflections of content are provided when participants adopt resistant behavior (eg, do not elaborate on their beliefs, choose to do no PA for a certain week).	When the Facebook moderator spots a participant who provided a comment demonstrating resistance talk, she reflects on the comment without arguing for change, in a way to demonstrate comprehension of the participant position. She concludes by asking an open question to the participant and the group, encouraging further self-exploration.
Explore options [2]	The intervention asks participants open and multiple choice questions during motivational sessions. [3]	The Facebook moderator asks participants open questions after the publication of web articles. [4]
Encourage Change-Talk [2]	Motivational sessions use MI interviewing skills (OARS; [4]) and other techniques oriented in favor of change.	Facebook moderator uses MI interviewing skills (OARS; [4]) and other techniques oriented in favor of change.
**SDT-related strategies**		
Provide a menu of effective options for change [5]	The action planning tool proposed preferred physical activities and solutions to common barriers of people with type 2 diabetes.	The fifth and ninth web articles shows preferred physical activities and solutions to common barriers of people with type 2 diabetes.
Provide a rationale for information given [5]	The rationale of each motivational session and tool is explained either through a video or short sentence on the first page of each intervention component.	The rationale of each intervention component is always explained through a short text provided at the beginning of a specific component. For example, the rational of each web article is provided in its first paragraph.

		Supporting patients' choices and initiatives [5]	Positive feedback is provided to participants when they make even the smallest commitment toward change. The intervention does not judge participants who provide answers that are not in keeping with change.	Positive feedback is provided to participants when they make a comment about even the smallest commitment toward change within the Facebook group. The intervention does not judge participants who provide answers that are not in keeping with change.
**Competence: feeling effective in one's ongoing interactions with the social environment and experiencing opportunities to exercise and express one's capacities [6]**				
**MI-related strategies**				
		Present clear and neutral information about behavior and outcomes [2]	Participants can receive information about the risks associated with physical inactivity and the benefits of regular physical activity during the first two motivational sessions. Participants are told explicitly that they are the only experts about what and how to change.	Participants can receive information about the risks associated with physical inactivity and the benefits of regular physical activity during the first two web articles and eleventh article. Participants are told explicitly throughout the intervention that they are the only experts about what and how to change.
		Help the client develop appropriate goals [2]	The action plan tool encourages participants to set realistic weekly behavioral goals for themselves.	The web article presenting the action plan tool encourages participants to set realistic weekly behavioral goals for themselves.
		Provide positive feedback [2]	Participants are frequently valorised for their participation in the motivational sessions and efforts toward change. Participants are also valorised for their strengths and values in motivational sessions 3 and 5.	Based on participants' comments following the publication of a web article, the Facebook moderator provides a summary of all the comments (for every web article) in which participants are valorised for their participation in the group and efforts toward change.
		Support self-efficacy [2]	The intervention affirms the strengths of participants and unconditionally recognizes their capacity and ability to change.	Affirmation of participants' strengths and unconditional recognition of their capacity/ability to change is explicitly mentioned in web articles and other intervention's components.
**SDT-related strategies**				
		Help skills building and problem solving [5]	The intervention gives participants information and tools on how to calculate and self-monitor their PA level. It also helps participant identify effective solutions to their barriers and provides information on how to practice PA safely.	The intervention gives participants information and tools on how to calculate and self-monitor their PA level. It also helps participant identify effective solutions to their barriers and provides information on how to practice PA safely.

**Relatedness: feeling connected to others, to caring for and being cared for by those others, to having sense of belongingness both with other individuals and with one's community [7]**				
**MI-related strategies**				
		Express empathy [2]	Messages are written in a way that shows participants that their opinions matter.	Interactions made by the Facebook group moderator are written in a way that shows participants that their opinions matter.
		Explore client's concern [2]	The first motivational session explores worries participants may have toward their insufficient PA level in an empathic way. A tab is dedicated to participants who want to share their opinion on the website or express concerns about it (same as *Technical support* tab).	The Facebook group moderator asks open questions allowing participants to express worries they may have toward their insufficient PA level. A tab is dedicated to participants who want to share their opinion on the website or express concerns about it (same as *Technical support* tab).
		Demonstrate understanding of the client's position [2]	Reflections and summaries are used throughout the motivational sessions to try to understand what participants think or feel.	Reflections and summaries are used within the Facebook group by the moderator to try to understand what participants think or feel.
		Avoid judgment or blame [2]	Messages to participants are written in a neutral or rewarding style.	Web articles and messages to participants are written in a neutral or rewarding style.
**SDT-related strategies**				
		Provide unconditional positive regard [5]	Messages to participants are never judgmental. The intervention unconditionally recognizes that participants are able to change their PA behavior.	Information provided to participants is never judgmental. The intervention unconditionally recognizes that participants are able to change their PA behavior.
		Provide a consistently warm interpersonal environment [5]	The introduction videos at the start of each motivational session use an enthusiastic but calm tone. We have tried to make the website and tools inviting and warm for participants based on feedback received during usability testing.	The writing style of the Facebook moderator and web articles attempted to be enthusiastic and positive. We have tried to make the website and tools inviting and warm for participants based on feedback received during usability testing.

REFERENCES

1. Deci, E.L. and R.M. Ryan, *Intrinsic Motivation and Self-Determination in Human Behavior*. 1985: Springer.
2. Markland, D., et al., *Motivational interviewing and self–determination theory.* Journal of social and clinical psychology, 2005. **24**(6): p. 811-831.
3. Friederichs, S.A., et al., *Motivational interviewing in a web-based physical activity intervention: questions and reflections.* Health Promot Int, 2013.
4. Miller, W.R. and S. Rollnick, *Motivational interviewing: Helping people change*. 2012: Guilford Press.
5. Patrick, H. and G.C. Williams, *Self-determination theory: its application to health behavior and complementarity with motivational interviewing.* Int J Behav Nutr Phys Act, 2012. **9**: p. 18.
6. Ryan, R.M. and E.L. Deci, *Self-determination theory and the facilitation of intrinsic motivation, social development, and well-being.* American psychologist, 2000. **55**(1): p. 68.
7. Ryan, R.M., *Psychological needs and the facilitation of integrative processes.* Journal of personality, 1995. **63**(3): p. 397-427.
