# Supplementary material for: Effectiveness of Computer Tailoring Versus Peer Support Web-Based Interventions in Promoting Physical Activity Among Insufficiently Active Canadian Adults With Type 2 Diabetes: Protocol for a Randomized Controlled Trial
Source: JMIR Res Protoc. 2016 Feb 11;5(1):e20. doi: 10.2196/resprot.5019 (PMC4768043; doi:10.2196/resprot.5019)
Supplement: Multimedia Appendix 3 [file resprot_v5i1e20_app3.pdf]

Multimedia Appendix 3.

Questions posted by the moderator into the Facebook group

simultaneously with the publication of each web article

Please consider before reading:

The questions and introduction text below have been translated from French to English. The French version was used for the intervention.

Web Article 1

- How much PA are you doing per week at the moment?
- How do you plan to reach the recommendations of the Canadian Diabetes Association?

Web Article 2

- What benefits related to physical activity are the most important to you?
- Specifically, how can you make sure to get these benefits?

Web Article 3

- How your environment can help you in your physical activity routine?
- What strengths that you possess could help you overcome the barriers preventing you to move more?

Web Article 4

- What would your action plan look like in terms of physical activity this week?
- Try to describe a plan as shown in the web article!

Web Article 5

- How an adjustment plan might be helpful to you?
- If it suits you, what would your adjustment plan look like?

Web Article 6

- What physical activities of moderate intensity could you practice this week?
- What would be your tricks to perform these activities regularly?

Web Article 7

- Which of these exercises meet your needs?
- How can you incorporate these exercises in your daily life?

Web Article 8

- How did this web article help you learn more about physical activity?
- How will you apply that knowledge in your everyday life?

Web Article 9

- What are your favorite physical activities?
- How will you go on using these to perform at least 150 minutes of moderate intensity physical activity per week?

Web Article 10

- What would be your strategies to stay active in the summer?
- What would be your strategies to stay active in the winter?

Web Article 11

- What are your biggest motivations for being regularly active?
- What are the elements giving you confidence in your ability to be active regularly?

Introduction text posted in
the description section of the Facebook group

The Diabète en Forme team welcomes you on your Facebook discussion group.

Some of you may have participated in other online group before. This group could be different. While discussing several topics related to physical activity, the main idea behind our group is to support you through your physical activity journey in the way that you will consider the more helpful to you. We suggest that each member of the group help other members with that same mentality.

The interest of this group and Diabète en Forme is to help each other and make everyone's life better. Let’s try together to do what is best for everyone, without forcing a person to change against his will, or without his permission. The objective of Diabète en Forme is actually not to tell you how your life could be better, because we believe that only you can really say what is best for you and what you need to change.

Based on these principles, we suggest 4 general rules for the group:

1. Confidentiality. Respect the confidentiality of information exchanged between members of the Facebook discussion group. Shared information and experiences must stay as much as possible inside the group, as well as within the study.

2. Respect. Sometimes, some members may feel vulnerable because of what they share. It is therefore important to show respect for the challenges and experiences of each member, as well as their efforts to move forward. Examples of disrespect are: interrupt, criticize unjustifiably, aggressively impose his opinions to others and ignore all about one person.

3. Alternately. The people in the group are not forced to speak on any subject. That said, let us ensure that everyone can express himself if he wants, leaving room for everyone to do so. Let’s use the strength of each member of this group. We encourage that all group discussions include all people wishing to take part in the conversation.

4. All equal. Although the moderator is responsible for advancing the group in the right direction, he is not above anyone. Everyone is also responsible for the ultimate goal of the group: helping each member to increase his day-to-day level of physical activity! Like you, the moderator is human. She will therefore answer to many comments posted in this group, but not all. She will finally interact for a limited time each day.

Without further ado, we invite you all to discuss together and move towards your goals!

Sincerely,

The Diabète en Forme team

END
