# Supplementary material for: Effectiveness of Computer Tailoring Versus Peer Support Web-Based Interventions in Promoting Physical Activity Among Insufficiently Active Canadian Adults With Type 2 Diabetes: Protocol for a Randomized Controlled Trial
Source: JMIR Res Protoc. 2016 Feb 11;5(1):e20. doi: 10.2196/resprot.5019 (PMC4768043; doi:10.2196/resprot.5019)

Multimedia Appendix
Websites' layouts

Fully-automated, tailored, Web-based intervention

Peer support, Web-based intervention

Log-in Page

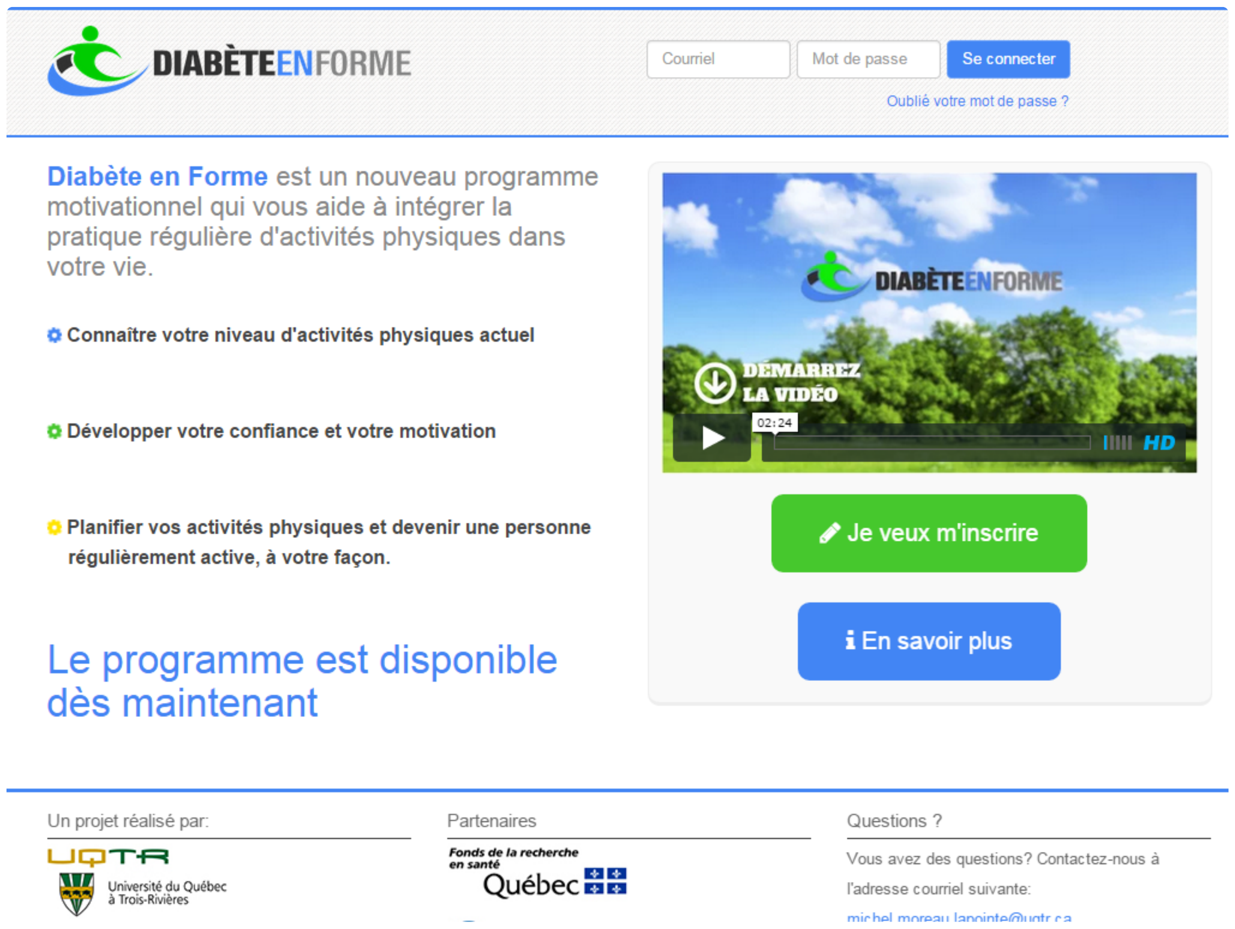

Log-in Page

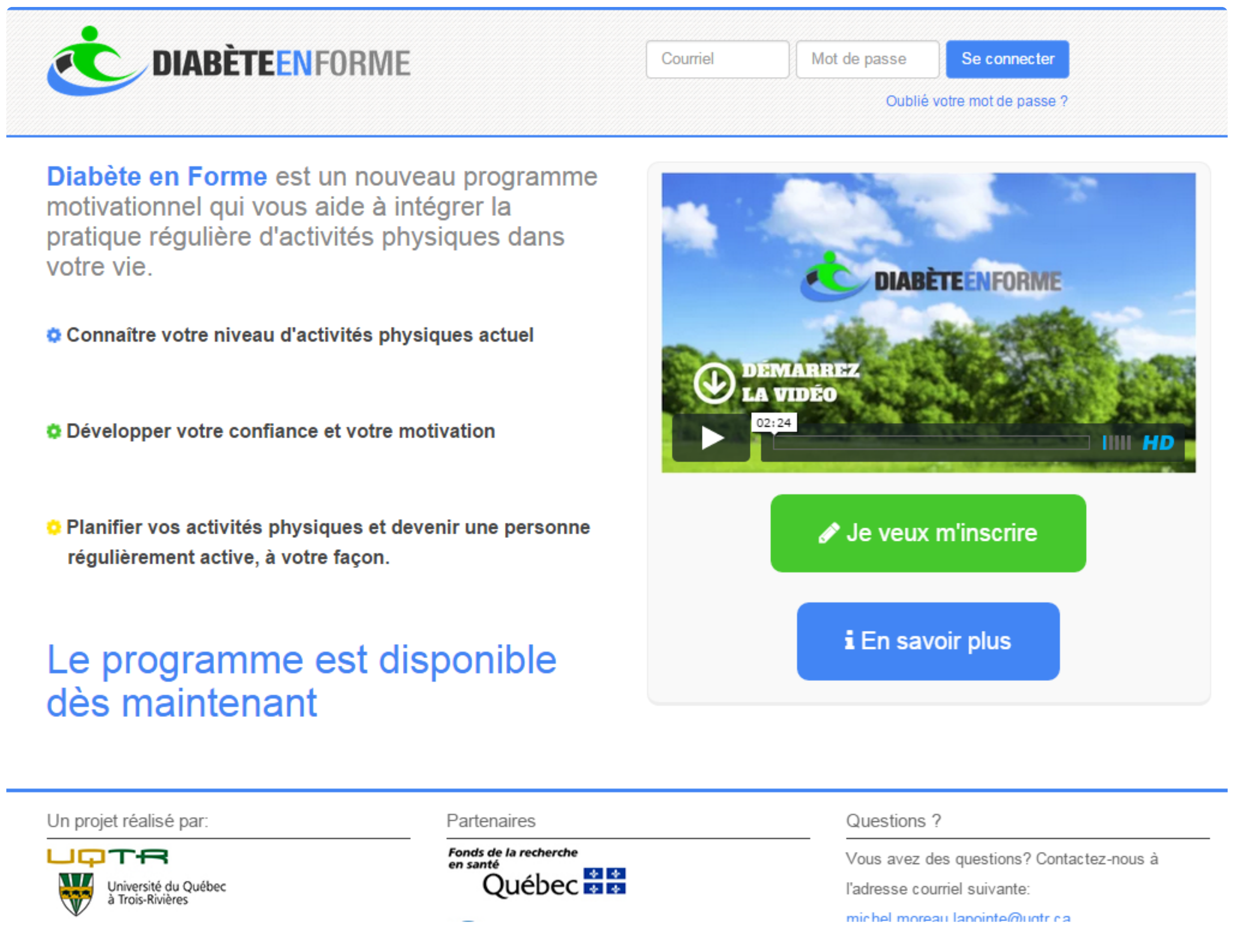

Home Page
(when logged in)

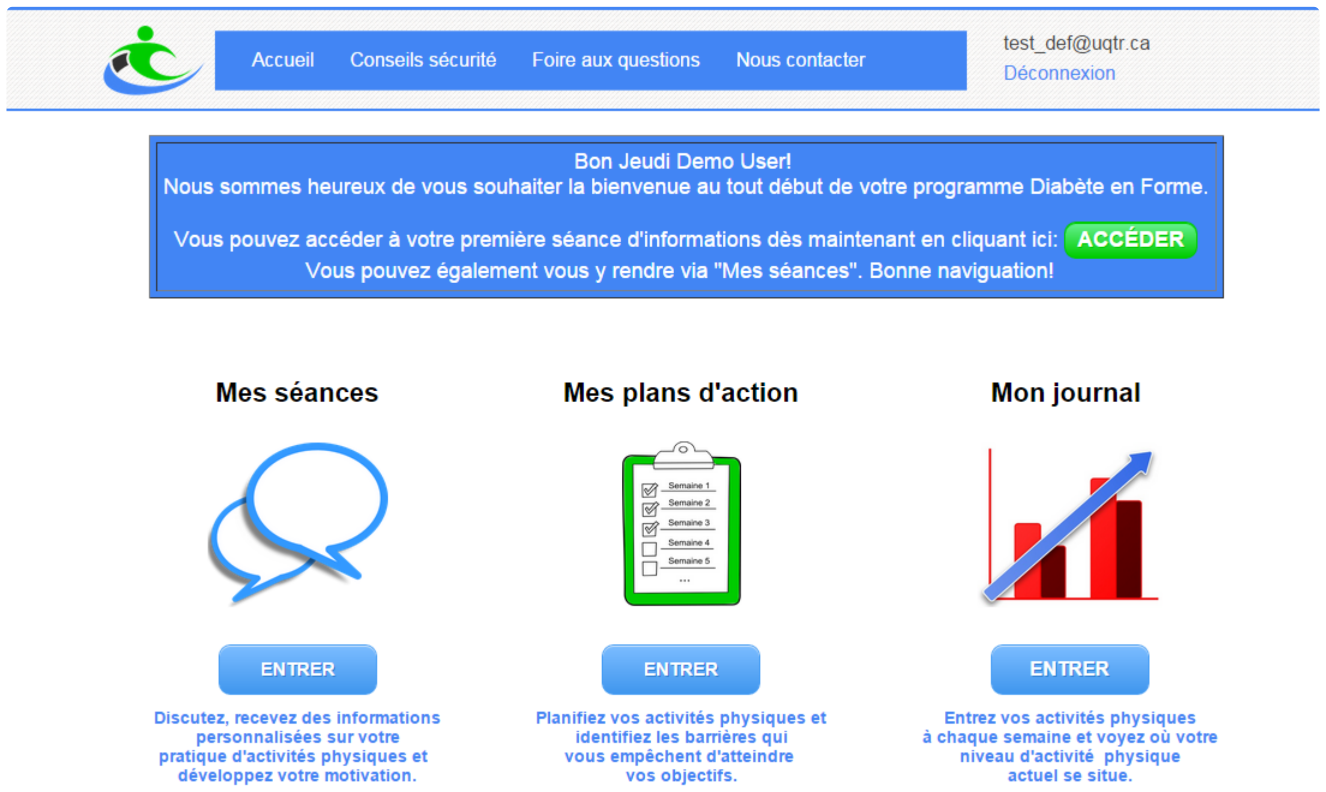

Home Page
(when logged in)

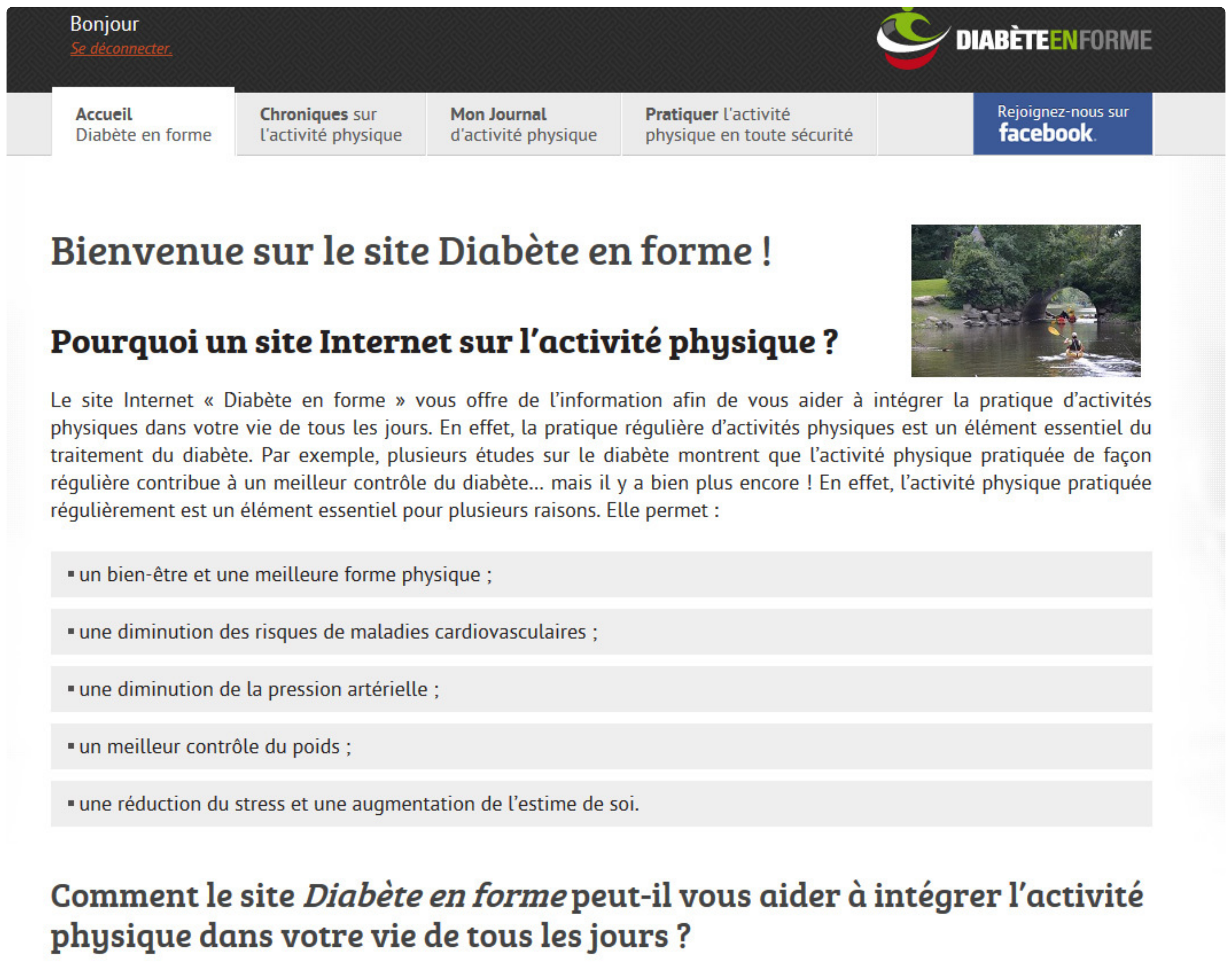

Supplement: Multimedia Appendix 4 [file resprot_v5i1e20_app4.pdf]
