# Supplementary material for: Effectiveness of Computer Tailoring Versus Peer Support Web-Based Interventions in Promoting Physical Activity Among Insufficiently Active Canadian Adults With Type 2 Diabetes: Protocol for a Randomized Controlled Trial
Source: JMIR Res Protoc. 2016 Feb 11;5(1):e20. doi: 10.2196/resprot.5019 (PMC4768043; doi:10.2196/resprot.5019)
Supplement: Multimedia Appendix 5 [file resprot_v5i1e20_app5.pdf]

**Multimedia Appendix 5.** Constructs targeted from the theoretical model of both interventions and their related evidence-based or theory-based behavior change techniques.

| **Constructs included in the computer-tailoring assessment[a]** | | |
|---|---|---|
| Construct & behavior change techniques | **Computer-tailored intervention** | **Peer-support intervention** |
| **Self-efficacy** | | |
| Reduce negative emotions [1] | The intervention provides tailored evaluative feedback[b] on self-efficacy that reassures participants with low self-efficacy scores. | The intervention recognizes unconditionally the capacity of a participant to practice regular PA within the web articles and Facebook group. |
| Self-talk [1] | At the end of an action-planning activity, the intervention encourages participants to persevere no matter what the final outcomes of the week's plan are. | Unused |
| Social support (unspecified)[c] [1, 2] | An MI-like session is provided after feedback on self-efficacy, allowing participants to select strengths that could help them change. An elaboration exercise follows where participants conduct a more in-depth evaluation of how those strengths could help them change concretely. | MI type of counselling is provided within the Facebook group of the intervention. Facebook moderator asked questions to know more about participants' strengths, and later reflects on the strengths participants have shared within the group. This takes place after the third web article. The moderator also explicitly recognizes participants' capacity to change. |
| Social support (practical) [1] | Unused | Participants have opportunities to exchange ideas on the way to perform a certain type of PA, or on any other technicalities related to PA. The moderator can also participate in this kind of conversations within the Facebook group. |
| Social support (emotional) [1] | Unused | Participants have opportunities to mutually support themselves through the struggles and successes associated to the journey toward being regularly active. The moderator can also emotionally |

| | | | | |
|---|---|---|---|---|
| | | | | support participants through the Facebook group. |
| | | Vicarious experiences [3] | Video testimonies of 2 regularly active people with type 2 diabetes are provided during the intervention's 6th tailored motivational session. | The Facebook moderator encourages participants to share their PA experiences and successes with others via the Facebook group. |
| | | Imaginary reward [4] | During the 6th tailored motivational session, participants are invited to explore what their ideal PA practice would be like 1 year after the intervention. They are also asked what they would gain from this accomplishment and how proud they would feel if they succeed at being regularly active within a year after the intervention. | Unused |
| | | Social reward [1] | During motivational sessions 4 and 8, participants are valorized by the intervention if they took part in more PA during the intervention than when they started. The self-monitoring tool praises participants with evaluative feedback[b] every time they participate in a new PA session. | Participants have opportunities to be praised by other participants or the moderator within the Facebook group when they share a PA accomplishment within the group. |
| | | Self-reward [1] | If the participant's current PA level is higher than their initial PA level during motivational sessions 4 and 8 (when PA level is reassessed), an evaluative feedback[b] is provided advising participants to be proud of themselves. | If a participant demonstrated that he increased his PA level during the intervention, the Facebook moderator will congratulate the participant and advise him that he can be proud of himself. |
| **Confidence ruler (computer-tailored intervention only, strictly for intervention purpose)** | | | | |
| | | Social support (unspecified) [c] [1, 2] | Tailored comparative progress feedback[b] is provided during motivational session 8; it compares the initial confidence ruler score with the current one. The feedback is accompanied by an MI question eliciting change-talk tailored to their current confidence ruler score. | Unused |
| **Attitude** | | | | |
| | | Information about health consequences [1-3] | Participants are informed on the consequences of an insufficient PA level during motivational | Participants are informed on the consequences of an insufficient PA level in |

| | | | | |
|---|---|---|---|---|
| | | | session 1 and on the benefits of an optimal PA level during motivational session 2. | the first web article and on the benefits of an optimal PA level in the second web article. |
| | | Reduce negative emotions [1] | The intervention provides tailored evaluative feedback[b] based on the overall attitude score that reassures participants when the score is low, and congratulates them when the score is high. | The second web article roll with resistance (MI strategy) by acknowledging that many people with type 2 diabetes possibly disregard the benefits of PA. On the other hand the same web article explains that the evidences are very clear about the PA benefits for people with type 2 diabetes. |
| | | Social support (unspecified) [c] [1, 2] | Two MI-like sessions are provided aiming at changing attitude of participants. Motivational session 1 allows participants to select two health-risks associated with type 2 diabetes that they want to avoid, which they later elaborate on to analyze what the concrete impacts of those risks in their lives would be. Similar activities are repeated during motivational session 2. This time, participants identify two PA benefits that are important to them instead of two health risks. | MI type of counselling is provided within the Facebook group of the intervention. Facebook moderator asked questions to know more about participants' most preferred PA benefits, and later reflects on the benefits participants have shared within the group. This takes place after the second web article. |
| | | Social support (practical) [1] | Unused | Participants have opportunities to mutually support themselves by exchanging information on the pros and cons of regular PA. The moderator can also participate in this kind of conversation through the Facebook group. |
| **Importance ruler (computer-tailored intervention only, strictly for intervention purpose)** | | | | |
| | | Social support (unspecified) [c] [1, 2] | Tailored comparative progress feedback[b] is provided during motivational session 4. This feedback compares the initial importance ruler score with the current one and is accompanied by an MI-based question eliciting change-talk tailored to their current importance ruler score. | Unused |
| **Intention** | | | | |

| | | | | |
|---|---|---|---|---|
| | | Social support (unspecified) [c] [1, 2] | Tailored comparative progress feedback[b] is provided during motivational sessions 4 and 8. It compares the initial intention score with the current one and is accompanied by an MI-based question eliciting change-talk. | Participants have opportunities to mutually support themselves by exchanging information on their most important motivations toward practicing regular PA. This takes place after the eleventh web article. The moderator can also participate in this kind of conversation through the Facebook group. |
| | | Social support (emotional) [1] | Unused | Participants have opportunities to mutually support themselves by acknowledging each other's motivation for doing regular PA. The moderator can also emotionally support participants on this subject through the Facebook group. |
| | | Reduce negative emotions [1] | The intervention provides tailored evaluative feedback[b] on the intention score; it reassures participants with a low score and congratulates participants with a high one. | The web article advises participants to not worry if they possess a low intention score and that the intervention is there to help them improve and feel more confident they can practice regular PA. |
| **PA behavior** | | | | |
| | | Feedback on behaviour [1, 5] | During motivational session 1, tailored comparative-normative feedback[b] comparing the participants' current PA level with the PA recommendations of the Canadian Diabetes Association is provided to participants. Participants can view their total minutes of moderate intensity PA per week, for each week of the intervention, via the self-monitoring tool. | Participants can view their total minutes of moderate intensity PA per week, for each week of the intervention, via the self-monitoring tool. |
| | | Review behavioral goal(s) [1, 2, 5] | During their action plan activities, participants are encouraged to choose a higher PA level than their current one for their weekly goals; however, we recommend setting a higher goal only if they feel confident enough to reach it. | During the web article 4 presenting the action plan tool, participants are encouraged to choose a higher PA level than their current one for their weekly goals; however, we recommend setting a higher goal only if they feel confident enough to reach it. |

| | | | | |
|---|---|---|---|---|
| | | Social comparison [1] | During motivational session 1, participants are informed about the percentage of Canadians with type 2 diabetes meeting PA recommendations versus those not meeting them. | During web article 1, participants are informed about the percentage of Canadians with type 2 diabetes meeting PA recommendations versus those not meeting them. |
| | | Social support (practical) [1] | Unused | See same BCT for Self-Efficacy |
| | | Instruction on how to perform the behavior [1, 3] | Participants are advised about what PA intensity to aim for in the first tailored motivational session. In addition, a tab is dedicated to safety advice for people with type 2 diabetes practicing PA. | Participants are advised about what PA intensity to aim for in the first web article. In addition, a tab is dedicated to safety advice for people with type 2 diabetes practicing PA. |
| | | Commitment [1] | At the end of the action plan tool, participants can click on a commitment check box that reads "I, (name of participant), commit to executing my action plan for the week". | Unused |
| | | Self-monitoring of behavior [1, 5-7] | A specific tool on the website is dedicated to participants who wish to monitor their behavior. | A specific tool on the website is dedicated to participants who wish to monitor their behavior. |
| | **Type of motivation** | | | |
| | | Social support (unspecified) [c] [1, 2] | During motivational session 3, the intervention provides descriptive feedback[b] on type of motivation whether participants possess more controlled or autonomous forms of motivation. During the same motivational session, participants are asked to identify their most important values to see how regular PA would help them be more "congruent" with these values in their daily lives. The intervention as a whole is also designed to develop an autonomous motivation toward the regular practice of PA in participants. | The intervention as a whole is designed to develop an autonomous motivation toward the regular practice of PA in participants. |
| **Constructs not included in the computer-tailoring assessment[a]** | | | | |
| **Awareness: Cues, knowledge, and risk perception** | | | | |
| | | Feedback on behavior [1, 5] | See Feedback on behavior for PA behavior. | See Feedback on behavior for PA behavior. |

| | | | | |
|---|---|---|---|---|
| | | Information about health consequences [1-3] | See Information about health consequences for Attitude. | See Information about health consequences for Attitude. |
| | | Prompt/cues [1] | When participants complete an action plan, they receive an email advising them that it is now time to execute it. In their action plan, participants also select the days that they will practice their physical activities. | In their action plan, participants selects the days that they will practice their physical activities. |
| **Social influence** | | | | |
| | | Social support (unspecified) [c] [1, 2] | Social support is provided through our MI-SDT concept application construct throughout the intervention activities (see Table 2). | Social support is provided through our MI-SDT concept application construct throughout the intervention activities (see Table 2). |
| | | Social support (practical) [1] | Unused | Opportunities for practical social support are discussed under previous specific constructs. |
| | | Social support (emotional) [1] | Unused | Opportunities for emotional social support are discussed under previous specific constructs. |
| | | Social comparison [1] | See Social comparison for PA behavior. | See Social comparison for PA behavior. |
| **Action plans—barriers** | | | | |
| | | Goal setting (behavior) [1, 5-7] | Participants are asked to set a behavioral goal (ie, a PA level they want to reach for a specific week) each time they build an action plan. | Participants are asked to set a behavioral goal (ie, a PA level they want to reach for a specific week) each time they build an action plan. |
| | | Problem-solving [1, 2] | During their action-planning activities, participants can select which barrier is more likely to prevent them from being active and then select a solution to overcome this barrier accordingly. | During their action-planning activities, participants can select which barrier is more likely to prevent them from being active and then select a solution to overcome this barrier accordingly. |
| | | Action planning [3, 8] | A specific tool on the website serves to build action plan for each week of the intervention. | A specific web article on the website serves to build action plan for each week of the intervention. It provides a tool they can use for each week of the intervention and also after the intervention. |
| **Personal factors** | | | | |

| | | | | |
|---|---|---|---|---|
| | | Content is personalized for each participant [9] | All emails and messages of the motivational sessions are adapted by gender (Contextualization[b]) and use the participants' names (Identification[b]). Participants are also explicitly advised that the intervention will give them feedback based on their answers to the tailoring assessment questionnaire (Raising expectations of customization[b]). | Sometimes, mainly for the last four weeks, participants can receive personalised feedback by the Facebook group moderator. |
| **Message factors** | | | | |
| | | Tailored messages adapted to participants' scores of the theoretical model constructs [9] | Tailored feedback is provided during each motivational session for constructs included in the tailoring assessment questionnaire. | Unused |
| **Channel factors** | | | | |
| | | Use of the Internet and emails [10] | Only website pages and emails are used to deliver messages to participants. | Only website pages and emails are used to deliver messages to participants. |
| **Source factors** | | | | |
| | | Credible source [11] | The team responsible for the DEF intervention is composed of exercise and diabetes experts. Each motivational session starts with a video featuring an exercise specialist who explains the purpose of each session. Home page video is also featuring an exercise specialist explaining the whole purpose of the intervention. | The team responsible for the DEF intervention is composed of exercise and diabetes experts. Each web article is signed with a credible figure at the end, entitled with "physical activity specialist". Home page video is also featuring an exercise specialist explaining the whole purpose of the intervention. |

[a]These subtitles only apply for the fully-automated, computer-tailored, Web-based intervention.
[b]The terminology used for the types of tailored feedback and other tailored components is based on the Harrington & Noar reporting standards for tailored interventions [12] .
[c]Motivational Interviewing techniques used as part of both interventions have been coded as *social support (unspecified)* because the behavior change techniques taxonomy used for this paper labels Motivational Interviewing as such [13]. Details about all motivational sessions and intervention overview are provided in Steps 2 and 4.

## REFERENCES


1. Olander, E.K., et al., *What are the most effective techniques in changing obese individuals' physical activity self-efficacy and behaviour: a systematic review and meta-analysis.* International Journal of Behavioral Nutrition and Physical Activity, 2013. **10**(1): p. 29.
2. Avery, L., et al., *Changing physical activity behavior in type 2 diabetes: a systematic review and meta-analysis of behavioral interventions.* Diabetes Care, 2012. **35**(12): p. 2681-9.
3. Ashford, S., J. Edmunds, and D.P. French, *What is the best way to change self-efficacy to promote lifestyle and recreational physical activity? A systematic review with meta-analysis.* Br J Health Psychol, 2010. **15**(Pt 2): p. 265-88.
4. Miller, W.R. and S. Rollnick, *Motivational interviewing: Helping people change*. 2012: Guilford Press.
5. Michie, S., et al., *Effective techniques in healthy eating and physical activity interventions: a meta-regression.* Health Psychol, 2009. **28**(6): p. 690-701.
6. Bird, E.L., et al., *Behavior Change Techniques Used to Promote Walking and Cycling: A Systematic Review.* 2013.
7. Broekhuizen, K., et al., *A systematic review of randomized controlled trials on the effectiveness of computer-tailored physical activity and dietary behavior promotion interventions: an update.* Ann Behav Med, 2012. **44**(2): p. 259-86.
8. Williams, S.L. and D.P. French, *What are the most effective intervention techniques for changing physical activity self-efficacy and physical activity behaviour--and are they the same?* Health Educ Res, 2011. **26**(2): p. 308-22.
9. van Stralen, M.M., et al., *The long-term efficacy of two computer-tailored physical activity interventions for older adults: main effects and mediators.* Health Psychol, 2011. **30**(4): p. 442-52.
10. Kohl, L.F., R. Crutzen, and N.K. de Vries, *Online prevention aimed at lifestyle behaviors: a systematic review of reviews.* J Med Internet Res, 2013. **15**(7): p. e146.
11. Forbes, C.C., et al., *Physical activity preferences and type 2 diabetes: exploring demographic, cognitive, and behavioral differences.* Diabetes Educ, 2010. **36**(5): p. 801-15.
12. Harrington, N.G. and S.M. Noar, *Reporting standards for studies of tailored interventions.* Health Educ Res, 2012. **27**(2): p. 331-42.
13. Michie, S., et al., *The behavior change technique taxonomy (v1) of 93 hierarchically clustered techniques: building an international consensus for the reporting of behavior change interventions.* Ann Behav Med, 2013. **46**(1): p. 81-95.
